# Psychometric evaluation of the L Test for functional mobility across EDSS-based severity levels in multiple sclerosis

**DOI:** 10.1007/s11845-026-04463-3

**Published:** 2026-05-25

**Authors:** Hanife Abakay, Mehmet Fatih Yetkin, Özge Baykan Çopuroğlu, Tuğba Dere

**Affiliations:** 1 Incesu Ayse and Saffet Arslan Health Services Vocational School, Kayseri University, Kayseri, Türkiye; 2 Department of Neurology, Faculty of Medicine, Erciyes University, Kayseri, Türkiye; 3 Sarıkaya School of Physiotherapy and Rehabilitation, Yozgat Bozok University, Yozgat, Turkey

**Keywords:** Functional mobility, L Test, Multiple sclerosis, Reliability, Validity

## Abstract

**Background:**

Functional mobility impairment is common in Multiple Sclerosis (MS) and affects daily functioning.

**Aim:**

The present study aimed to examine the validity and reliability of the L Test, a measure commonly used to assess functional mobility, across different levels of disease severity in individuals with MS.

**Methods:**

A total of 35 individuals with clinically confirmed MS and Expanded Disability Status Scale (EDSS) scores ranging from 0–3.5 were recruited. Participants with EDSS scores between 0 and 1.5 were assigned to Group 1, while those with scores between 2–3.5 constituted Group 2. To determine inter-rater reliability, the L Test was administered on the same day by two independent physiotherapists.

**Results:**

Group 1 comprised 17 participants with a mean age of 40.82 ± 13.62 years, while Group 2 included 18 participants with a mean age of 48.44 ± 8.89 years. The test–retest reliability of the L Test was excellent, with an intraclass correlation coefficient (ICC) of 0.99 (95% CI: 0.999–1.000). Receiver operating characteristic (ROC) analysis demonstrated that the L Test had a very high discriminative ability in differentiating between the two groups (AUC = 0.997). Correlation analyses revealed a strong, positive, and statistically significant relationship between the L Test and the TUG Test in both groups (Group 1: r = 0.712, *p* = 0.001; Group 2: r = 0.856, *p* < 0.001).

**Conclusion:**

The findings indicate that the L Test is a valid and reliable tool for assessing functional mobility in individuals with MS presenting with varying functional levels.

## Introduction

Multiple sclerosis (MS) is a chronic, inflammatory, and autoimmune disorder of the central nervous system [1]. The clinical spectrum of MS is remarkably broad, encompassing motor, sensory, cognitive, and emotional domains. Among its numerous manifestations, gait disturbance stands out as one of the earliest and most frequently observed clinical signs since the historical descriptions of the disease [2]. Impairments in walking and balance can emerge even during the initial stages of MS and progressively lead to marked reductions in mobility and overall quality of life [3]. Beyond limiting independence, these impairments substantially elevate the risk of falls approximately 40% of individuals with MS are reported to experience fall-related injuries [4, 5].

Accurate assessment of functional mobility using valid and reliable instruments is therefore essential for identifying real-world performance deficits and designing individualized rehabilitation interventions. In clinical practice, several standardized functional tests such as the Timed Up and Go (TUG), the Timed up and down stairs (TUDS) test, and the L Test are frequently employed for this purpose [6–8]. These tests provide a practical means to evaluate balance, turning ability, transfer capacity, and lower-limb function, thus contributing substantially to both clinical decision-making and the evaluation of therapeutic efficacy.

The Expanded Disability Status Scale (EDSS) remains the most widely utilized measure for quantifying disease severity and disability in MS. The scale offers a comprehensive overview of neurological functioning and mobility status, thereby reflecting disease progression across multiple domains [9]. Prior studies have demonstrated that increases in EDSS scores are typically accompanied by noticeable deterioration in walking and balance performance [10]. However, direct comparisons of the validity and reliability of functional mobility tests across varying EDSS levels remain limited. This lack of evidence highlights a gap in the literature regarding the sensitive detection of functional impairments during the early stages of MS.

In this context, the present study aimed to investigate the validity and reliability of the L Test in individuals with MS whose EDSS scores were 3.5 or lower and to explore differences in mobility across disease severity groups. A distinctive aspect of this study is the evaluation of the L Test not only in comparison to healthy controls but also among individuals with MS representing different levels of disability. This approach allows for a more comprehensive understanding of the test’s applicability across varying clinical presentations. The findings are expected to enhance early detection of subtle functional deficits, support clinicians in developing personalized rehabilitation strategies, and provide a robust methodological foundation for future interventional research.

## Methods

### Participants

This study was conducted with individuals aged 18–45 years who had a confirmed diagnosis of MS. Participants were recruited from the MS outpatient clinic of the Department of Neurology at Erciyes University Hospital. A total of 35 individuals who met the inclusion criteria were enrolled.

Prior to data collection, ethical approval was obtained from the Institutional Ethics Committee of Erciyes University (Approval No: 156/2024, Date: 21.11.2024). The study was carried out in accordance with the ethical principles of the Declaration of Helsinki. All participants were fully informed about the study’s purpose, procedures, potential benefits, and possible risks, and provided written informed consent prior to participation.

Participants were stratified into two groups based on their EDSS scores to classify disease severity. This grouping was intended to capture functional differences and create homogeneous subgroups for analysis. Individuals with normal neurological examination findings (EDSS 0–1.5) were assigned to Group 1, while those with mild to moderate disability (EDSS 2–3.5) were assigned to Group 2.

Sample size was calculated based on a large effect size (*d* = 1.18) obtained from a previous reference study. Assuming a 95% confidence level and 95% statistical power, a minimum of 34 participants was determined to be required [11].

Inclusion criteria were as follows: a confirmed diagnosis of MS by a neurologist, age between 18 and 45 years, the ability to maintain an upright standing position independently, an EDSS score of ≤ 3.5, and adequate cognitive capacity to comprehend instructions (Mini-Mental State Examination score > 24).

Exclusion criteria included experiencing a relapse within the previous 30 days, the presence of any additional neurological, orthopedic, or systemic disorder that could affect gait or balance, and pregnancy in female participants. Individuals who were unable to follow instructions or presented with severe visual or cognitive impairment were also excluded. These criteria were established to ensure the reliability and validity of the functional mobility assessments.

## Assessment tools

### Expanded disability status scale (EDSS)

The EDSS is a standardized tool that quantifies disability on a scale from 0 (normal) to 10 (death due to MS). It evaluates eight functional systems—pyramidal, cerebellar, brainstem, sensory, bowel and bladder, visual, and cerebral functions. Higher scores indicate greater disability. Scores between 0–1.5 indicate no disability, 2–3.5 mild, 4–6.5 moderate, and 7–10 severe disability [12].

### L Test of functional mobility

The L Test is a modified version of the TUG test designed to provide a more comprehensive assessment of functional mobility. It requires the participant to stand up from a chair, walk along an L-shaped 20-m pathway, and return to the seated position. The test incorporates two transfers and four directional changes. Chair height was adjusted so that each participant’s feet rested flat on the floor and both hips and knees were flexed at approximately 90°. Participants began the test on the verbal cue “start,” followed the marked route, and returned to the starting position. Total time was recorded in seconds [7].

To assess reliability, the same physiotherapist administered the L Test twice during the first session, separated by a one-hour interval. One week later, the test was repeated by a second physiotherapist using the same standardized procedure. Thus, the study design allowed for the evaluation of both intra-rater reliability (same physiotherapist, same day) and inter-rater reliability (different physiotherapists, one-week interval), rather than representing a pure test–retest reliability design.

### TUG test

Participants were seated on a standard chair (seat height: 46 cm) with their backs supported and feet fully in contact with the floor. After receiving detailed instructions, each participant was asked to stand up on the verbal cue “start,” walk a marked distance of three meters at a comfortable pace, turn around, return to the chair, and sit down again. The total time required to complete the sequence was measured in seconds using a stopwatch [6].

### TUDS test

This test was used to evaluate lower-limb strength, balance, and functional mobility. Each participant stood at the base of a staircase consisting of ten steps, each 17 cm high. On the command “start,” participants ascended the stairs step by step to the top platform, turned around, and descended in the same manner to the starting point. A handrail was available for safety but was used only when necessary. Timing began with the “start” cue and stopped once the participant’s feet returned fully to the floor at the starting position [8].

### Statistical analysis

All statistical analyses were performed using SPSS version 25.0 (IBM Corp., Armonk, NY, USA). Continuous variables were expressed as mean ± standard deviation or median (interquartile range: 25th–75th percentiles), and categorical variables as frequency and percentage. The Shapiro–Wilk test was used to assess data normality.

When parametric assumptions were satisfied, group differences were analyzed using independent-samples t-tests; otherwise, the Mann–Whitney U test was applied. Categorical variables were compared using the chi-square test. Pearson or Spearman correlation coefficients were calculated to explore associations between continuous variables.

The consistency of the L Test was evaluated using the intraclass correlation coefficient (ICC), minimal detectable change (MDC), standard error of measurement (SEM), and Bland–Altman plots. Receiver operating characteristic (ROC) curve analysis was performed to determine the diagnostic accuracy and discriminative performance of the L Test. Statistical significance was set at *p* < 0.05 [13].

## Results

No statistically significant differences were found between the groups in terms of age, height, weight, or body mass index (BMI) (*p* > 0.05). However, significant between-group differences were observed for the L Test, TUG, TUDS test, MS subtype distribution, and EDSS scores (p < 0.05). L Test times were significantly higher in Group 2 compared to Group 1, indicating poorer functional mobility in individuals with greater disability. Similarly, both TUG and TUDS test durations were significantly longer in Group 2 than in Group 1.

Regarding disease subtype, the proportion of Relapsing Remitting MS (RRMS) was higher in Group 1, whereas the proportion of Primary Progressive MS (PPMS) was significantly greater in Group 2. EDSS scores were also significantly higher in Group 2, confirming greater disability severity (Table 1).

**Table 1:** Demographic and clinical characteristics of the PwMS

		Group 1		Group 2		
		X̄ ± SD	Med (IQR)	X̄ ± SD	Med (IQR)	p
Age (years)		40.82 ± 13.62	45 (30–53.5)	48.44 ± 8.89	50 (43—55)	0.062 (t = −1.948)
Height (cm)		164.59 ± 10.32	162 (155–175.5)	165.28 ± 6.44	165.5 (160.75–170.5)	0.59 (z = −0.546)
Weight (kg)		73.59 ± 13.08	75 (64.5—82.5)	69.72 ± 9.92	73 (58.5—78)	0.546 (z = −0.629)
BMI (kg/m^2^)		27.27 ± 5.09	25.65 (23.96—29.64)	25.51 ± 3.23	26.97 (23.2—27.67)	0.228 (t = 1.228)
L test		14.31 ± 3.15	13.8 (11.7—16.5)	30.13 ± 6.22	30.25 (24.73—35.35)	0.0001* (t = −9.574)
TUG		8.59 ± 9.35	5.9 (5.2—7.65)	16.11 ± 4.2	16 (13.05—19.1)	0.0001* (z = −4.457)
TUDS		12.8 ± 10.11	8.2 (7.8—13.61)	36.83 ± 42.17	26.95 (23.66—29.53)	0.0001* (z = −4.358)
MS clinic type	Relapsing Remitting	12 (%70.6)		3 (%16.7)		0.003* (cs = 123.740)
	Secondary Progressive	4 (%23.5)		6 (%33.3)		
	Primary Progressive	1 (%5.9)		8 (%44.4)		
	Progressive Relapsing	0 (%0)		1 (%5.6)		
EDSS score		1.41 ± 0.26	1.5 (1.25—1.5)	3.32 ± 0.25	3.5 (3—3.5)	0.0001* (z = −5.201)

*p < 0.05 statistically significant difference; *t* Independent samples t test; *z* Mann Whitney U test; *cs* Chi-square statistic X̄ ± SD Mean ± Standard Deviation, *BMI* Body mass index; *Med* Median; *IQR* Interquartile range; *TUG* Timed up and go; *TUDS* Timed up and down stairs; *EDSS* Expanded disability status scale; *PwMS* People with multiple sclerosis

A moderate and statistically significant positive correlation was found between age and L Test performance in Group 1, whereas no significant correlation was detected in Group 2. BMI was positively and moderately correlated with L Test duration in Group 1 but not in Group 2.

Strong positive correlations were observed between the L Test and the TUG in both groups, indicating that longer L Test durations were associated with slower TUG performance. Additionally, a strong positive and statistically significant correlation was found between the L Test and the TUDS test in Group 1; however, this relationship did not reach statistical significance in Group 2. No significant correlations were found between L Test results and height, weight, or EDSS scores in either group (Table 2).

**Table 2:** Correlations between the L Test and other outcome measures in PwMS

		L test	
		Group 1	Group 2
Age (years)	r	0.672^*pr^	0.072^pr^
	p	0.003	0.778
Height (cm)	r	−0.424^sp^	−0.067^pr^
	p	0.089	0.791
Weight (kg)	r	0.325^pr^	0.022^sp^
	p	0.204	0.930
BMI (kg/m^2^)	r	0.637^*pr^	0.006^pr^
	p	0.006	0.981
TUG	r	0.712^*sp^	0.856^*pr^
	p	0.001	0.000
TUDS	r	0.846^*sp^	0.438^sp^
	p	0.0001	0.069
EDSS Scores	r	−0.256^sp^	−0.013^sp^
	p	0.322	0.962

*p < 0.05 statistically significant correlation; *pr* Pearson correlation coefficient; *sp* Spearman correlation coefficient; *pr* Pearson correlation coefficient; *TUG* Timed up and go; *TUDS* Timed up and down stairs; *EDSS* Expanded disability status scale

The mean change in L Test time was –0.366 ± 0.492 s. The ICC was 0.999 (95% CI: 0.999–1.000). indicating excellent test–retest reliability. The MDC₉₅ was calculated as 0.23 s. representing the smallest detectable difference that can be considered clinically meaningful. The standard error of measurement (SEM₉₅) was 0.016. demonstrating a minimal degree of measurement error (Table 3).

**Table 3:** Test–retest reliability of the L Test in PwMS

	Change mean (SD)	ICC (95% CI)	95% CI of the Difference		MDC_95_	SEM_95_
			Lower	Upper		
L test	−0.366 (0.492)	0.999 (0.999–1.000)	−0.535	−0.197	0.23	0.016

*ICC* Intraclass correlation coefficient; *MDC95* Minimal detectable change (MDC95 = 1.96 × SEM95 × √2); *SEM95* Standard error of measurement [SEM95 = SD × √(1-ICC)]; *SD* Standard deviation; *CI* Confidence interval

**Fig. 1:**
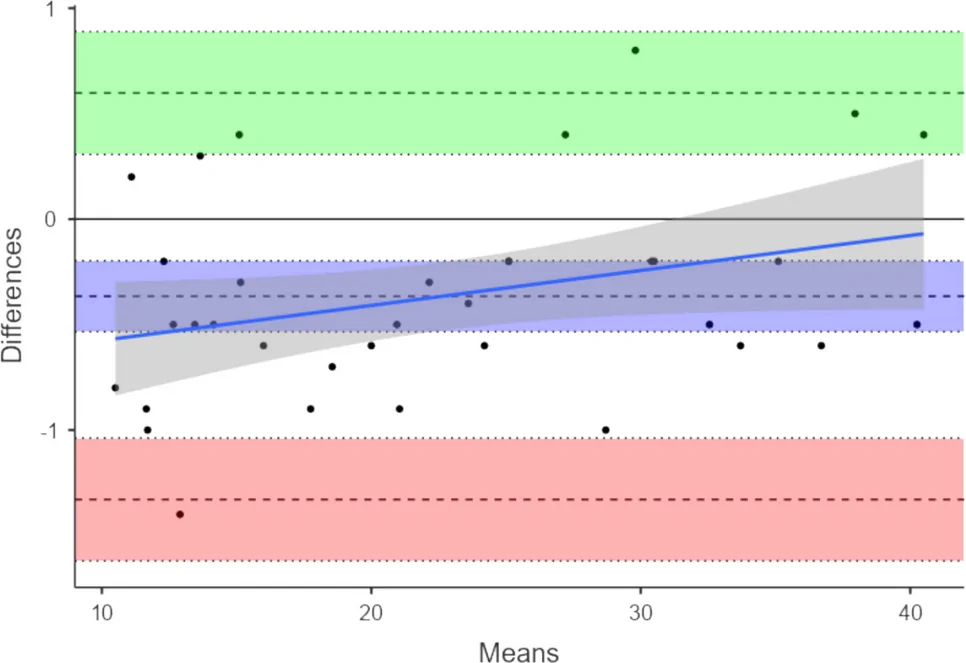
Bland–Altman analysis showing agreement between repeated L Test measurements

The Bland–Altman analysis demonstrated excellent agreement between repeated L Test measurements (Fig. 1).

**Fig. 2:**
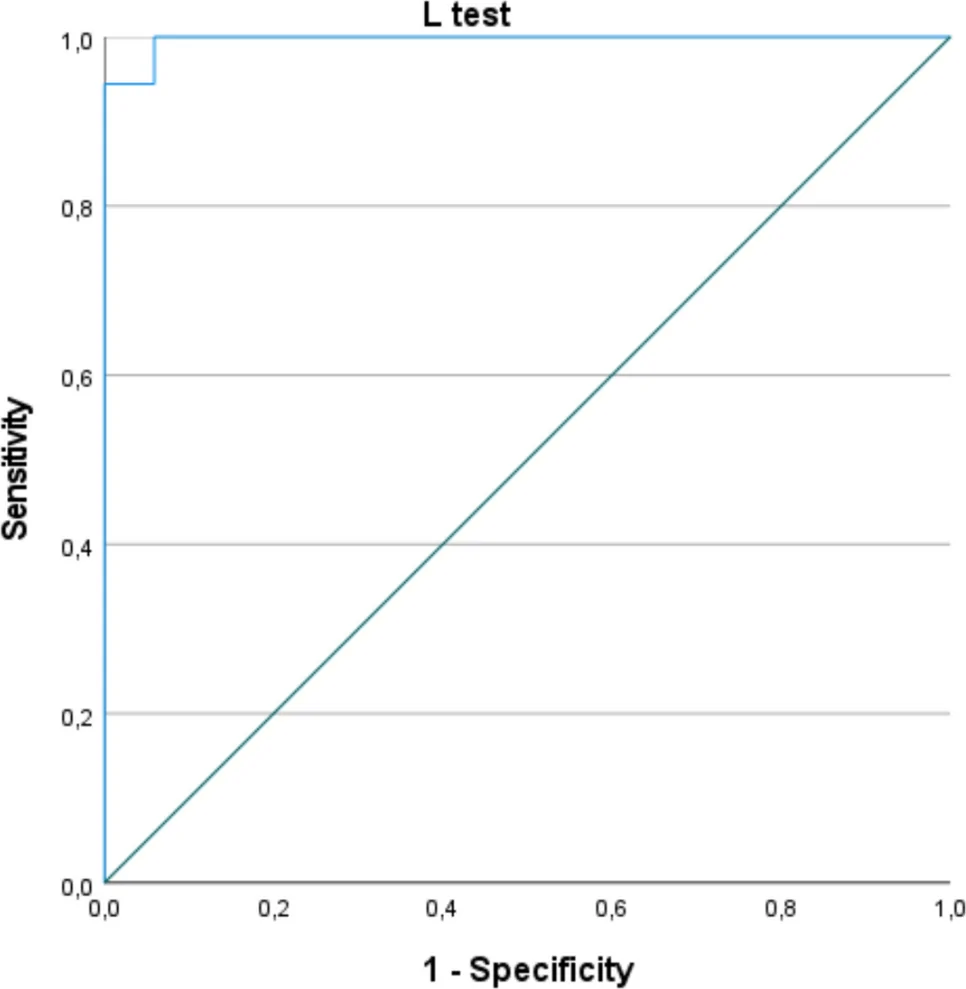
Receiver operating characteristic (ROC) curve for discrimination of EDSS-based functional levels using L Test scores

ROC curve analysis revealed that the L Test possessed outstanding discriminative ability for distinguishing between the two EDSS-based groups. with an area under the curve (AUC) value of 0.997 (*p* < 0.0001) (Fig. 2).

## Discussion

This study aimed to evaluate the validity and reliability of the L Test, a functional mobility assessment widely used in neurorehabilitation among individuals with MS. The L Test demonstrated excellent reliability (ICC = 0.99) and strong discriminative ability between disability levels (AUC = 0.997). Strong positive correlations were observed with the TUG test in both groups, while correlations with the TUDS test were significant only in Group 1. No significant associations were found with EDSS scores, suggesting that the L Test may better reflect real-world functional capacity rather than neurological impairment alone. The observed MDC (0.23 s) and SEM (0.016) indicate minimal measurement error.

The absence of significant demographic differences suggests that variations in functional performance were primarily attributable to disease type and severity rather than age or anthropometric factors. This aligns with previous literature indicating that demographic factors, particularly BMI, have a limited impact on mobility, whereas disease progression and neurological involvement are major determinants of functional capacity [14].

The L Test has been widely employed across neurological populations. Beyond MS and stroke, its reliability and validity have been confirmed in individuals with Parkinson’s disease and post-stroke patients. In post-stroke populations, the test demonstrated excellent inter- and intra-rater reliability (ICC ≈ 0.99) with an MDC of approximately four seconds [15]. Similarly, in Parkinson’s disease, the L Test showed strong correlations with the TUG and 10-Meter Walk Test, reflecting its ability to assess both mobility and functional independence [16].

Recent MS studies further support these findings. Bilek and Demir (2024) reported excellent test–retest reliability (ICC = 0.996) and strong correlations with TUG and 10-MWT in 82 individuals with MS [17]. Eldemir et al. [18] examined 34 MS patients (EDSS 0–5) and 34 healthy controls, reporting ICC = 0.995 and MDC = 1.4 s, with positive correlations between the L Test, TUG, and 360° Turn Test, and negative correlations with 10-MWT and 6-MWT [18].

This study extends prior research by comparing L Test performance across different EDSS levels and MS clinical forms, directly linking disease stage with functional performance. Additionally, the correlation between the L Test and the TUDS test was explored for the first time. The ICC value obtained in the present study was 0.999. This extremely high ICC value indicates excellent test–retest reliability of the measurement. However, this result may have been influenced by methodological factors such as the homogeneity of the sample, limited variability in the measured variable, and the implementation of highly standardized procedures among raters. The reported MDC value (0.23 s) indicates that the measurement has high sensitivity and can reliably detect even small changes. However, the clinical relevance of such a low MDC value should be carefully considered by clinicians. Although minimal measurement error provides a statistically robust threshold for change, whether this change represents a clinically meaningful improvement should be interpreted within the context of the clinical characteristics of the studied population and the functional relevance of the measured variable. Therefore, the clinical applicability of the MDC should be considered not only on a statistical basis but also in conjunction with other functional and clinical outcome measures. ROC analysis confirmed the test’s excellent discriminative ability (AUC = 0.997); however, this very high value should be interpreted with caution due to the relatively small and homogeneous sample and the limited EDSS range. Future studies with larger and more heterogeneous populations are warranted to validate these findings.

The significantly higher EDSS scores in Group 2 were consistent with poorer functional test performance, supporting the established role of EDSS as a key clinical index of disease severity that integrates ambulation and motor function [19]. The lack of a significant relationship between the L Test and EDSS scores suggests that the EDSS may not fully reflect all clinical components influencing functional mobility performance. The EDSS is primarily based on neurological examination findings, functional system involvement, and particularly ambulation capacity [9]. However, it may demonstrate limited sensitivity to aspects such as fatigue, dynamic balance, turning performance, transfer abilities, proprioceptive deficits, higher-level walking tasks, and cognitive-motor dual-task performance, all of which affect mobility in daily life. In contrast, the L Test assesses multi-component functional tasks including sit-to-stand transfers, walking, directional changes, and turning. Therefore, it may reveal performance differences among individuals with similar EDSS scores. In addition, since EDSS scoring is based on clinician judgment, it may not always fully capture symptom fluctuations or the patient’s functional status on a given day. For this reason, performance-based measures are considered to provide complementary information to the EDSS [9, 20, 21]. Functional tests such as the L Test and TUG not only assess mobility but also provide insights into independence in daily activities, balance, and fall risk. Identifying the interaction between functional performance, disease stage, and clinical subtype is essential for targeted rehabilitation and longitudinal follow-up.

Taken together, these results suggest that the L Test is a reliable, valid, and discriminative tool for assessing functional mobility in individuals with MS. Its ease of administration, sensitivity to functional changes, and strong psychometric properties make it valuable for both clinical practice and research. The comparison with widely used measures such as TUG and TUDS enhances the clinical interpretability and applicability of the findings. The elevated standard deviation observed in some variability measures of the TUG test further supports the presence of heterogeneity in functional mobility levels within the sample. This variability may be attributed to differences in the severity of demyelination and neurological involvement in individuals with MS, leading to heterogeneous impairments in gait performance, balance control, and motor response speed.

## Strengths and limitations

Major strengths of this study include the comprehensive evaluation of reliability, validity, and discriminative performance, the use of multiple advanced statistical approaches (ICC, SEM, MDC, Bland–Altman, and ROC analysis), and stratification of participants according to EDSS scores, which allowed for a nuanced understanding of functional mobility across the MS spectrum.

Limitations include the relatively small and homogeneous sample, the restricted EDSS range (≤ 3.5), cross-sectional single-center design, and potential confounding factors such as comorbidities and fatigue. These limitations may affect generalizability and causal interpretation. Future longitudinal studies with larger and more diverse samples are recommended to confirm these findings and examine the sensitivity of the L Test to disease progression and rehabilitation outcomes.

## Conclusions

The L Test is a reliable, valid, and sensitive tool for assessing functional mobility in individuals with MS. Clinically, its ease of administration, short completion time, and good patient tolerance make it a practical option for routine evaluations. Given that functional capacity and mobility are commonly impaired in MS, the L Test can be employed to monitor disease progression, evaluate rehabilitation effectiveness, and identify individuals at increased risk of falls. Its strong correlations with complementary measures such as TUG and TUDS further support the integration of these assessments for a comprehensive evaluation of functional mobility.

## Data Availability

The data supporting the findings of this study are available from the corresponding author upon reasonable request.
